# A High Performance Sensor for Triaxial Cutting Force Measurement in Turning

**DOI:** 10.3390/s150407969

**Published:** 2015-04-03

**Authors:** You Zhao, Yulong Zhao, Songbo Liang, Guanwu Zhou

**Affiliations:** The State Key Laboratory for Manufacturing Systems Engineering, Xi’an Jiaotong University, No. 28, Xianning West Road, Xi’an 710049, China; E-Mails: zhaoyou319@stu.xjtu.edu.cn (Y.Z.); coffeelsb@stu.xjtu.edu.cn (S.L.); alainzhou@stu.xjtu.edu.cn (G.Z.)

**Keywords:** accuracy, natural frequency, triaxial, cutting force, sensor, high speed, turning

## Abstract

This paper presents a high performance triaxial cutting force sensor with excellent accuracy, favorable natural frequency and acceptable cross-interference for high speed turning process. Octagonal ring is selected as sensitive element of the designed sensor, which is drawn inspiration from ring theory. A novel structure of two mutual-perpendicular octagonal rings is proposed and three Wheatstone full bridge circuits are specially organized in order to obtain triaxial cutting force components and restrain cross-interference. Firstly, the newly developed sensor is tested in static calibration; test results indicate that the sensor possesses outstanding accuracy in the range of 0.38%–0.83%. Secondly, impacting modal tests are conducted to identify the natural frequencies of the sensor in triaxial directions (*i.e.*, 1147 Hz, 1122 Hz and 2035 Hz), which implies that the devised sensor can be used for cutting force measurement in a high speed lathe when the spindle speed does not exceed 17,205 rev/min in continuous cutting condition. Finally, an application of the sensor in turning process is operated to show its performance for real-time cutting force measurement; the measured cutting forces demonstrate a good accordance with the variation of cutting parameters. Thus, the developed sensor possesses perfect properties and it gains great potential for real-time cutting force measurement in turning.

## 1. Introduction

Cutting force is a key factor that reflects metal cutting condition, such as workpiece quality, cutting power as well as tool wear in metal cutting operation [1,2,3,4,5]. By measuring real-time cutting forces, cutting condition is observed and the cutting parameters can properly be adjusted in time, which will in turn improve cutting efficiency, extend tool life and decrease machining cost. With the development of machining technique in terms of high precision and high speed, it becomes a growing demand of measuring cutting forces during the high-speed metal cutting process with high precision. Thus, a cutting force sensor with high accuracy and high natural frequency is in great demand.

Research about cutting force measurement has been carried on for many years since the middle of twentieth Century [6]; and different kinds of cutting force sensors have been created, such as current, vibrant, fiber-optical, strain gauge, piezoelectric sensors, *etc.* [7,8,9,10]. However, few of them can satisfy the requirement of high accuracy and high natural frequency. To date, much more effort is dedicated to strain gauge and piezoelectric sensors because of their high stability and favorable performance. For example, Ergun Ates and Kadir Aztekin developed a one piece lathe sensor for measuring cutting forces in two axes based on strain gauge [11]. The sensor possesses favorable measuring error (≤3.75%), but it was only capable of detecting two of the three cutting force components and the author did not clarify the cross-interference between the measured cutting forces. With the same principle, Tulio Hallak Panzera developed a triaxial cutting force sensor with its repeatability less than 8.4% [12]. However, the natural frequency is neglected in his paper. What is more, the developed sensor was not compatible with a lathe system because a lathe tool post has to be removed for installing the sensor. This may affect the original lathe system. Süleyman Yaldiz introduced a strain gauge type cutting force sensor for turning, too [13]. However, his work concentrated on low cross-sensitivity (0.17%–0.92%) and low output errors (0.12%–0.8%) while sacrificing its natural frequency (159.2 Hz). G. Totis and M. Sortino proposed a modular-piezoelectric sensor for triaxial cutting force measurement in turning [14]. This work exhibits a good performance with its static relative errors less than 5.91% and its natural frequency was about 1 kHz, according to the finite element method (FEM) calculation. Chao Wang, *et al.* designed a piezoelectric film embedded smart cutting tool for measuring cutting force that achieved good linearity and low cross sensitivity [15], but it can only measure one cutting force component and it is incapable of measuring constant force signals. Generally speaking, few cutting force sensors possess favorable accuracy and high natural frequency that can meet the requirement of measuring cutting forces under high-precision and high-speed metal cutting condition.

Considering the features mentioned above, a triaxial cutting force sensor with high accuracy, good compatibility and high natural frequency is proposed in this paper. Three main steps are taken to achieve the objective of high accuracy and high natural frequency. Firstly, a novel structure of two mutual-perpendicular octagonal rings is introduced as a sensitive element of the designed sensor; secondly, locations of strain gauges are specially selected under the guidance of circular ring theory and finite element method (FEM) simulation result; finally, twelve strain gauges are specifically organized into Wheatstone full bridge circuits for measuring triaxial cutting force components and attenuating cross-interference. Static calibration and dynamic cutting experiment are undertaken with high accuracy equipment to evaluate comprehensive performance of the sensor. Test results indicate that the developed sensor is qualified to measure cutting forces in high speed cutting process.

## 2. Experimental Section

### 2.1. Design Principle

**Figure 1:**
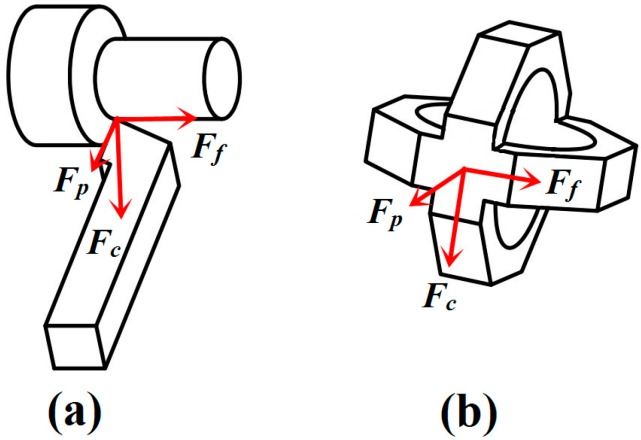
(**a**) Triaxial cutting force components; (**b**) Structure of two mutual-perpendicular octagonal rings.

The purpose of this study is to develop a high accuracy sensor capable of measuring triaxial cutting forces (*viz.* main cutting force *F_c_*, feeding force *F_f_* and thrust force *F_p_*; see Figure 1) during high speed turning process. A novel structure of two mutual-perpendicular octagonal rings was put forward for measuring triaxial cutting forces as depicted in Figure 1b. An octagonal ring is selected as sensitive element of the designed sensor due to the inspiration of circular ring theory as below [16]:

(1) $$\text{σ}=\text{α}F_{p}$$

(2) $$\text{σ}=\text{β}F_{c}$$

(3) $$\text{α}=\frac{\pi \left(k+1\right)\left(6r-t\right)sin\text{θ}-12r}{2\pi bt^{2}\left(k+1\right)}$$

(4) $$\text{β}=\frac{\left(\pi cos\text{θ}-4sin\text{θ}\right)t+6r\left(4sin\text{θ}-\pi cos\text{θ}-\pi \right)}{2\pi bt^{2}}$$

(5) $$k=\frac{r}{t}ln\left[\frac{r/t+0.5}{r/t-0.5}\right]-1$$

**Figure 2:**
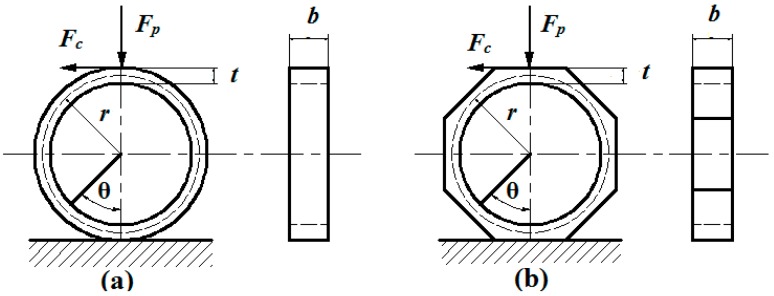
(**a**) Schematic view of a circular ring under the effect of *F_p_* and *F_c_*; (**b**) Schematic view of an octagonal ring under the effect of *F_p_* and *F_c_*.

For a circular ring, as illustrated in Figure 2a, when force *F_p_* or *F_c_* is applied to it, the outer surface stress of the ring caused by *F_p_* or *F_c_* can be calculated by Equations (1)–(5); Where, *σ* denotes surface stress of circular ring; α, β, and *k* are constants about ring parameters (*i.e.*, *r*, *t*, and *b*); *r*, *t*, and *b* represent circular ring’s mean radius, thickness and width as illustrated in Figure 2; *F_p_* and *F_c_* are vertical and tangential forces that applied on the top of the ring; θ denotes the inclination angle between the ring’s cross section and the vertical plane; and *π* is circumference ratio. Equations above indicate that ring’s surface stress is proportional to force *F_p_* and *F_c_*. As a circular ring is difficult to fix and it gives too little rigidity in *F_c_* direction, it is usually replaced by an octagonal ring because of similarity [17,18,19].

**Figure 3:**
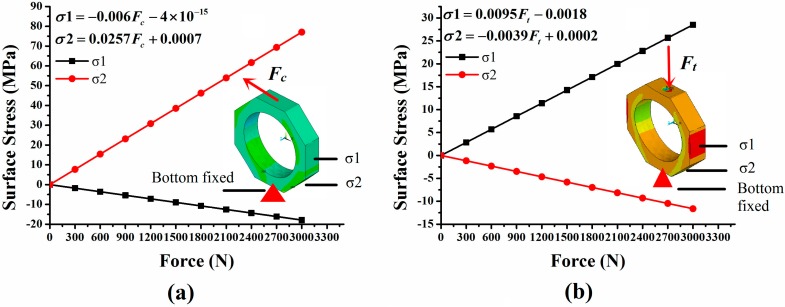
(**a**) FEM result for octagonal ring under the effect of *F_c_*; (**b**) FEM result for octagonal ring under the effect of *F_p_*.

As it is difficult to obtain theory formula to calculate the surface stress of an octagonal ring, FEM is invited to investigate the stress distribution of octagonal ring under the effect of *F_p_* or *F_c_*. We choose AISI1045 steel as the material of the simulated model in the FEM simulation process; its elastic modulus and Poisson ratio are 2.1 GPa and 0.269. The size of the octagonal model is set as: *t* = 6 mm, *r* = 30 mm and *b* = 15 mm. The element type used in this simulation is “solid92” and the volume mesh size of the model is 5 mm. Figure 3a shows the FEM result of the octagonal ring under the effect of *F_c_*, where σ*1* and σ*2* represent the stress at the center point of the octagonal ring’s outer surfaces as shown in Figure 3a. Figure 3b shows the FEM result of octagonal ring under the effect of *F_p_*, where σ*1* and σ*2* represent the same positions as mentioned above. It is obvious that the outer surface stress of octagonal ring is in good linear relationship with the applied force *F_p_* and *F_c_* as shown in Figure 3, this verifies that octagonal ring can be properly used as sensitive element in this study. What’s more, when *F_p_* is applied to octagonal ring, the stress distribution is symmetrical on the left and right half rings; the inner and outer surface stress on each half ring are equal and opposite in sign. For the octagonal ring under the effect of *F_c_*, stress distribution on the left and right half rings are equal and opposite in sign, and so are the inner and outer surface stresses on each half ring. This information is of great use in measuring circuit organization and cross-interference limitation.

### 2.2. Sensor Design and Fabrication

**Figure 4:**
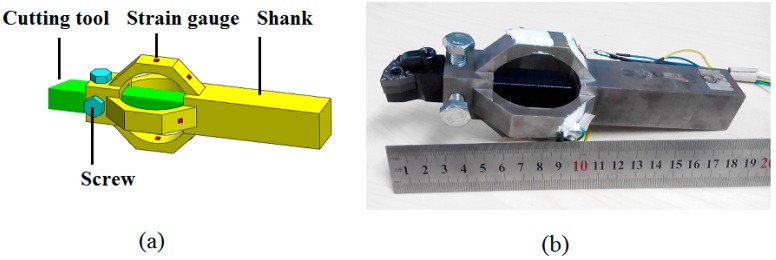
(**a**) Diagrammatic sketch of the designed sensor; (**b**) Photograph of the fabricated sensor.

A triaxial cutting force sensor for high accuracy and high natural frequency was designed as depicted in Figure 4a. The sensor includes three parts: part 1 is a slot with two tapping holes for fixing the cutting tool, which satisfies the interchangeability between different type of machine tools; part 2 is two mutual-perpendicular octagonal rings with strain gauges attached for separating, sensing and generating triaxial cutting force signals; and the third part is a shank for fixing the sensor on the tool post. The designed sensor is compatible with most lathe system because it can be installed without affecting original system.

**Figure 5:**
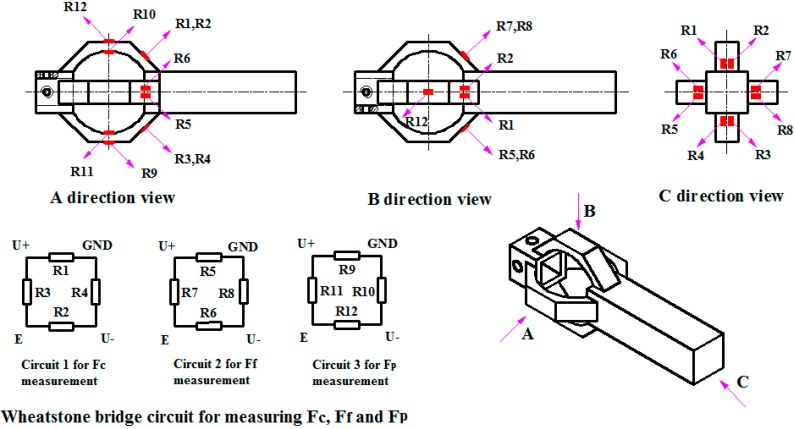
Locations of strain gauges and organization of Wheatstone full bridge circuits.

In this study, BF1000-1.5-90/BA type strain gauge is used as transduction element to convert surface stress into voltage signal. Twelve strain gauges are needed to form three Wheatstone full bridge circuits for measuring triaxial cutting forces as shown in Figure 5, and circuits 1, 2 and 3 are designed for measuring *F_c_*, *F_f_* and *F_p_*, respectively. For each circuit, the output voltage can be expressed as Equation (6):

(6) $$U=U_{+}-U_{-}=\left(\frac{R_{i}}{R_{i}+R_{i+2}}-\frac{R_{i+3}}{R_{i+1}+R_{i+3}}\right)E=\left[\frac{R+\Delta r_{i}}{\left(R+\Delta r_{i}\right)+\left(R+\Delta r_{i+2}\right)}-\frac{R+\Delta r_{i+3}}{\left(R+\Delta r_{i+1}\right)+\left(R+\Delta r_{i+3}\right)}\right]E$$

where *E* denotes input voltage of the Wheatstone circuit; *R_j_* (*j = i*, *i +* 1, *i +* 2, *i +* 3) represents resistors in each circuit and their resistance is *R*; ∆r*_j_* (*j = i*, *i +* 1, *i +* 2, *i +* 3) is resistance variation of *R_j_* under the effect of cutting force; *i* = 1, 5 and 9.

The sensor was fabricated into a monolithic piece with AISI 1045 steel by turning, milling, wire-electrode cutting as well as electric discharge machining. The surfaces of the rings were then ground for better strain gauge application. After that, strain gauges were mounted on the rings and three Wheatstone full bridge circuits were organized. Finally, the strain gauges were covered by silicone rubber type GD-414 to protect them from collision and corrosion. Figure 4b is a picture of the fabricated sensor.

### 2.3. Static Calibration

**Figure 6:**
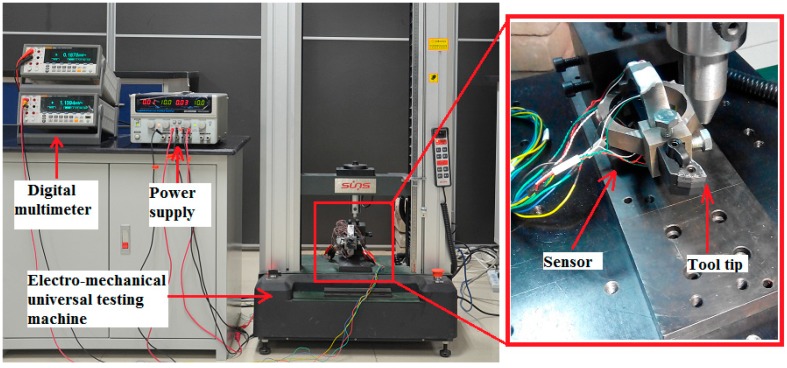
Experimental setup for static calibration.

Static calibration test was performed to determine the static properties of the sensor in three directions, namely *F_c_*, *F_f_* and *F_p_*, respectively. In this study, measuring range of each cutting force component was expected as 0~2000 N. Static calibration test was operated on an electro-mechanical universal testing machine which could apply standard force on the tool tip for a certain time. During the test, each cutting force component was applied from 0 N to its maximum value with an interval of 200 N; and each interval would be maintained for 30 s. The measuring circuits were excited by 10 V DC and the output signals were recorded by high accuracy (0.0025%) and resolution (6-1/2) precision digital multimeter. A schematic view of the experiment setup is shown in Figure 6.

### 2.4. Natural Frequency Identification

**Figure 7:**
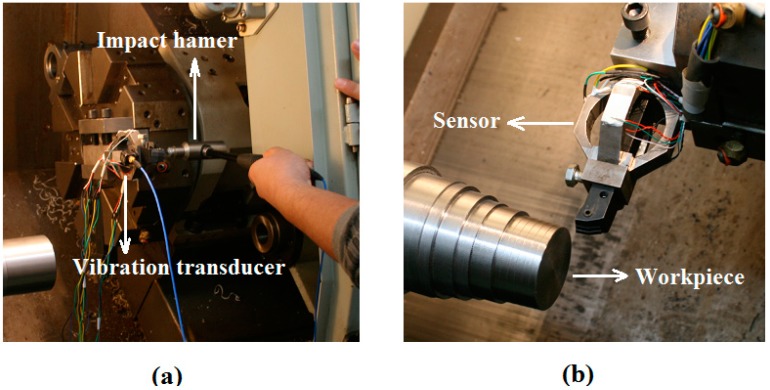
Experimental setup for: (**a**) impacting modal test and (**b**) dynamic cutting test.

Considering the fact that machine tool suffers from vibrations in certain cutting operation, documents suggest that the natural frequency of a sensor should be four times larger than the frequency of exciting vibrations in order to avoid these vibrations affecting the precision of the sensor [12,17,20,21]. Thus, it is necessary to determine the natural frequency of the sensing system after the sensor’s installment [14]. Identification of the natural frequency of the sensing system was accomplished by impacting modal test, as illustrated in Figure 7a. The sensor was installed on a NC lathe system (type FTC-20) with a triaxial vibration transducer (type 95663) connected to it. The instrumented sensor system was excited by an impact hammer (type 086D05) at a chosen excitation point, one direction at a time. For each direction of excitation, both the hammer’s signal and the vibration signal of the sensor system were acquired and processed by a mobile data acquisition system (type SCADAS305) from LMS Company (Leuven, Belgium).

### 2.5. Sensor Application in Dynamic Cutting Test

In this section, an application of the sensor during the turning process demonstrates its potential for real-time cutting force measurement (Figure 7b).

In detail, the experiment was operated on a NC lathe (type FTC-20), and a 95° of cemented carbide turning tool (type MWLNR1616H08) was selected. The workpiece used in this test was AISI1045 steel. Two groups of cutting tests were carried out; during group one, the cutting speed and depth of cut were kept constant while the feed rate varied from 0.1 mm/rev to 0.5 mm/rev in intervals of 0.05 mm/rev with time; in group two, the cutting speed and feed rate were kept constant while the depth of cut decreased from 1.1 mm to 0.1 with a 0.2 mm interval. More detailed information of the experiment parameters and equipment used in this paper can be found in Table 1 and Table 2, respectively.

**Table 1:** Experiment parameters of turning operation.

Group One			
Spindle speed (rev/min)	Diameter of the metal bar (mm)	Depth of cut (mm)	Feed rate (mm/rev)
300	63.0	0.5	0.10,0.15,0.20,0.25,0.30,0.35,0.40,0.45,0.50
**Group Two**			
Spindle speed (rev/min)	Diameter of the metal bar (mm)	Feed rate (mm/rev)	Depth of cut (mm)
300	62.0	0.2	1.1,0.9,0.7,0.5,0.3,0.1

**Table 2:** Detail information of the software and equipment involved in this paper.

Equipment	Type	Maker	Country
Strain gauge	BF1000-1.5-90/BA	SAISUO Electronic Technology Co. Ltd	Baoji, Shaanxi, China
Silicon rubber	GD-414	Bluestar New Chemical Material Co. Ltd	Chengdu, Sichuan, China
Electro-mechanical universal testing machine	UTM6104	Shenzhen Suns Technology Stock Co. Ltd	Shenzhen, Guangdong, China
Power supply	GPS-3303C	GWINSTEK Electronic Technology Co. Ltd	Suzhou, Jiangsu, China
Digital multimeter	8846A	FLUKE CORPORATION	USA
Mobile data acquisition system	SCADAS305	LMS Company	Belgium
NC lathe	FTC-20	Fair Friend Group	Taiwan, China
ANSYS software	ANSYS 12.1	ANSYS, Inc.	USA
Cutting tool	MWLNR1616H08	Birinai precision CNC group Limited	Taiwan, China

In order to predict theoretical cutting force trends during metal cutting, a classical mechanistic-empirical cutting force model was adopted as follow [22]:

(7) $$\begin{array}{l} F_{c}=9.81C_{F_{c}}\times {a_{p}}^{X_{F_{c}}}\times f^{Y_{F_{c}}}\times {\left(60v_{c}\right)}^{Z_{F_{c}}}\times K_{mF_{c}}\times K_{rF_{c}}\times K_{\lambda F_{c}}\times K_{\gamma_{\varepsilon }F_{c}}\\ F_{f}=9.81C_{F_{f}}\times {a_{p}}^{X_{F_{f}}}\times f^{Y_{F_{f}}}\times {\left(60v_{c}\right)}^{Z_{F_{f}}}\times K_{mF_{f}}\times K_{rF_{f}}\times K_{\lambda F_{f}}\times K_{\gamma_{\varepsilon }F_{f}}\\ F_{t}=9.81C_{F_{t}}\times {a_{p}}^{X_{F_{t}}}\times f^{Y_{F_{t}}}\times {\left(60v_{c}\right)}^{Z_{F_{t}}}\times K_{mF_{t}}\times K_{rF_{t}}\times K_{\lambda F_{t}}\times K_{\gamma_{\varepsilon }F_{t}} \end{array}$$

where *C_Fi_* (*i* *=* *c,f,t*) are constant coefficients about workpiece material and turning condition; *X_Fi_*, *Y_Fi_*, and *Z_Fi_* (*i* *=* *c,f,t*) are exponents for depth of cut (*a_p_*), feed rate (*f*) and cutting speed (*v_c_*), respectively; *K_mFi_*, *K_rFi_*, *K_λFi_*, and *K_γFi_* (*i* *=* *c,f,t*) are correction coefficients for material’s rigidity, tool cutting edge angle, tool cutting edge inclination angle and tool orthogonal rake angle, respectively. Both Equation (7) and these coefficients are summarized from practical cutting test under different cutting conditions and parameters. As Equation (7) is an empirical model extracted from practical cutting test, it cannot be used to accurately compute cutting forces in all metal cutting conditions, but it can be reliably adopted for predicting cutting force trend. Thus, Equation (7) is introduced to evaluate the performance of the sensor by comparing the measured cutting forces with the estimated ones calculated by Equation (7).

Table 3 tabulates coefficients for calculating cutting forces in this experiment, concerning AISI1045 and 95° of cemented carbide turning tool (MWLNR1616H08), except that *K_rFi_*, *K_λFi_*, and *K_γFi_* (*i* *=* *c,f,t*) are obtained by linear interpolation according to other tool angle coefficients because there is no coefficients for 95° of cemented carbide turning tool in [22].

**Table 3:** Cutting force coefficients for the mechanistic-empirical model.

	*C_Fi_*	*X_Fi_*	*Y_Fi_*	*Z_Fi_*	*K_mFi_*	*K_rFi_*	*K_λFi_*	*K_γFi_*
***F_c_* (*i = c*)**	270	1.0	0.75	−0.15	0.75	0.86	1.0	1.04
***F_f_* (*i = f*)**	294	1.0	0.5	−0.4	1.0	1.25	1.10	1.19
***F_p_* (*i = p*)**	199	0.9	0.6	−0.3	1.35	0.38	0.71	1.19

## 3. Results and Discussion

### 3.1. Static Calibration

The static calibration test for each cutting force component was performed five times and the output data of the sensor were averaged. In order to evaluate static properties of the devised senor, definition of corresponding indicators should be given first:

Hysteresis: for a certain input, the difference between output of the sensor in loading and offloading process is called hysteresis. Hysteresis error can be calculated as Equation (8); where “*H*” represents hysteresis error; “∆*y_max_*” is the sensor’s biggest output deviation between loading and offloading process under a series of input values; “*y_max_*” and “*y_min_*” are maximum and minimum output values of the sensor.

(8) $$H=\pm \frac{\Delta y_{\max}}{2\left(y_{\max}-y_{\min}\right)}\times 100\%$$

Repeatability: during the same working condition and within a short period, when input signals vary from the beginning to the end in full scale, the deviation among some groups of measured output values under the same input values is called repeatability. The repeatability error can be calculated by Equations (9) and (10). As for a certain test cycle, assuming the number of measuring points is *m*, and for each measuring point the output value has been measured for *n* times. Thus, “*S_i_* (*i* *=* 1*~m*)” stands for standard deviation of each measuring point; “*y_ij_*” is the *j*th (*j* *=* 1*~n*) measured value of the *i*th (*i* *=* 1*~m*) measuring point; “*y_i_*” is the average value of the measured values of *i*th measuring point. “*R*” stands for repeatability error; “*S_max_*” represents the biggest standard deviation among “*S_i_* (*i* *=* 1*~m*)”; “*y_max_*” and “*y_min_*” are maximum and minimum output values of the sensor.

(9) $$S_{i}=\sqrt{\frac{\sum_{j=1}^{n}{\left(y_{ij}-\bar{y_{i}}\right)}^{2}}{n-1}}$$

(10) $$R=\frac{3S_{\max}}{y_{\max}-y_{\min}}\times 100\%$$

Linearity: linearity is the biggest deviation between sensor’s practical output curve and its theoretical one. In this study, the sensor’s theoretical output curve is obtained by Least-square linear fitting method. Thus, the linearity error can be calculated as Equation (11); where “*L*” represents the linearity error; “∆*max*” is the biggest deviation value between the sensor’s practical output curve and its theoretical one.

(11) $$L=\frac{\Delta_{\max}}{y_{\max}-y_{\min}}\times 100\%$$

Accuracy: the accuracy of the sensor is a comprehensive indicator that reflects the sensor’s static performance; it can be calculated as Equation (12); where, “*A*” represents the accuracy error.

(12) $$A=\sqrt{H^{2}+R^{2}+L^{2}}$$

**Figure 8:**
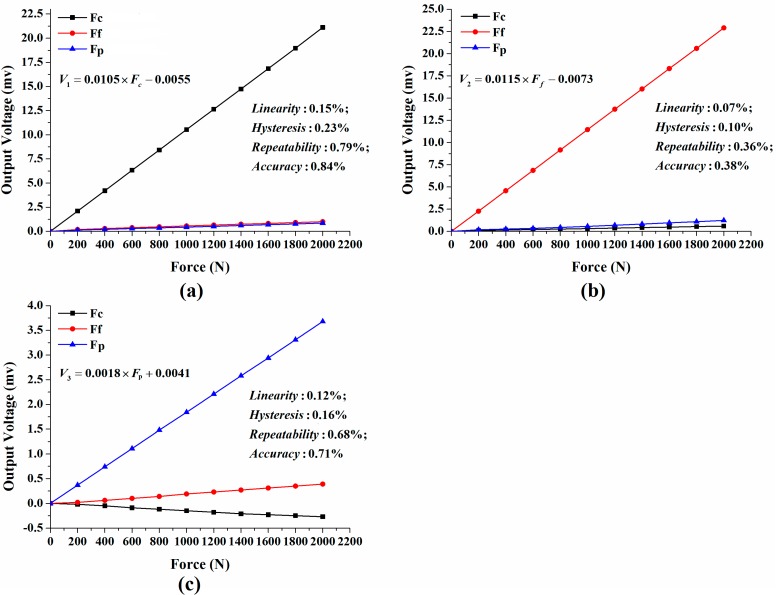
Calibration curve and cross-interference for: (**a**) main cutting force *F_c_*; (**b**) feed force *F_f_*; (**c**) thrust force *F_p_*.

Figure 8 depicts the calibration result for *F_c_*, *F_f_* and *F_p_*, respectively. As shown in the figure, the developed sensor possesses favorable static properties with all indicators less than 1%, which implies that it can satisfy the goal of high accuracy (≤0.84%).

The experimental sensitivity of the sensor signals (un-amplified) are, respectively, measured as 1.06 × 10^−2^ mv/N, 1.14 × 10^−2^ mv/N and 0.18 × 10^−2^ mv/N for *F_c_*, *F_f_* and *F_p_* through calibration test. It is apparent that the sensor’s sensitivity in *F_p_* direction is much smaller than the other two directions. This is believed to be caused by the sensor’s structure. According to the simulation result in Section 2.1, surface stress of the position for measuring *F_p_* is smaller than the one for *F_c_* (or *F_f_*) when an equal force *F_c_* (or *F_f_*) is applied to the octagonal ring, because *F_p_* is supported by two octagonal rings while *F_c_* and *F_f_* are supported by only one, thus the sensitivity of *F_p_* direction would be smaller than the other two directions in some extent.

**Figure 9:**
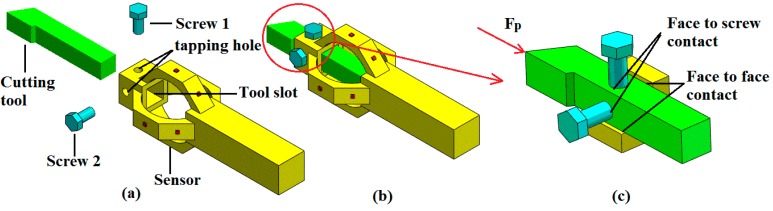
(**a**) Schematic view of the sensor before assemblage; (**b**) Schematic view of the sensor after assemblage; (**c**) Simplified view of the fixing method between the cutting tool and the sensor.

Cross-interference, defined as the ratio of sensor output in lateral axes to the one when force in primary axis, is an important factor for precise applications requiring high accuracy. The cross-interference of the sensor in *F_c_* and *F_f_* directions is in the range of 2.6%~5.4%, while the one in *F_p_* direction is 7.3%~10.5%. In this study, cross-interference is mainly thought to be caused by the eccentricity of tool tip. As illustrated in Figure 4, the tool tip is not on the axis of the sensor; thus, extra moment occurs when each cutting force component is applied on the tool tip. The extra moment leads to extra output in each cutting force measuring circuit. The fixing method between cutting tool and the senor results in the higher cross-interference in *F_p_* direction than the other two directions. In order to clearly display the fixing method of the cutting tool, a simplified view of sensor assemblage is provided in Figure 9. As the cutting tool and the sensor are connected by screws, they are not a monolithic piece; thus, connection clearance exists more or less between the tool and the sensor. What is more, the contact areas of the tool’s flank faces are different from each other (one is face to face, like the contact between bottom face of the tool and bottom face of the tool slot; another is face to screw, like the contact between the tool’s top face and screw 1), and they are not symmetric in structure. Thus, when thrust force *F_p_* is applied to the tool tip, the force distributes asymmetrically on the four half octagonal rings and makes the effect that *F_p_* cannot be offset in *F_c_* and *F_f_* measuring circuits, which makes the cross-interference more outstanding in *F_p_* direction.

### 3.2. Natural Frequency Identification

**Figure 10:**
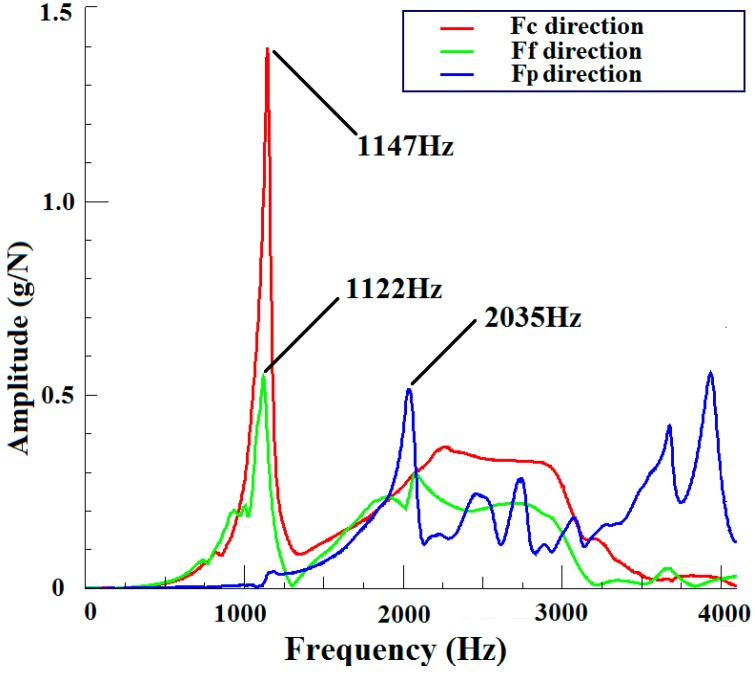
Frequency and amplitude result of impacting modal test.

Figure 10 shows the amplitude-frequency curves of impacting modal test. Ignoring the influence of low frequency disturbance, the sensor’s natural frequencies are 1147 Hz, 1122 Hz, 2035 Hz in *F_c_*, *F_f_* and *F_p_* directions, respectively. In turning process, the vibration frequency of machine tool is closely related to spindle speed; moreover, spindle rotating direction coincides with *F_c_* direction. Hence, the devised sensor can fulfill real-time cutting force measurement when spindle speed is less than 1147 × 0.25 × 60 = 17,205 rev/min; 17,205 rev/min is an impressive value in high speed cutting process. It is worth mentioning that this approach for computing the maximum spindle speed is mainly suitable for continuous cutting condition; other cutting conditions (e.g., interrupted cutting) are out of our research scope in this paper.

A comprehensive comparison is made between previously published designs and the sensor in this work. Whereas the static errors in [14,17,23] are expressed as a percentage of effective value, the static errors in [9,15,21] are expressed with respect to full-scale output (FSO). By analyzing the data listed in Table 4, the conclusion is made that the proposed design obtains a more favorable comprehensive property than previous works regarding the accuracy, cross-interference and natural frequency together.

**Table 4:** Comparison between the current state-of-the-art sensors and the sensor in this work.

Sensor Type	Linearity (%)			Cross-Axis Interference (%)			Natural Frequency (Hz)			Reference
	*F_c_*	*F_f_*	*F_t_*	*F_c_*→*F_f_* *F_t_*	*F_f_*→*F_c_* *F_t_*	*F_t_*→*F_c_* *F_f_*	*F_c_*	*F_f_*	*F_t_*	
Triaxial	1.4	1.3	1.2	9.2, 1.7	0.7, 1.8	0.5, 3.3	/	/	/	[17,23]
Monoaxial	4.6			5.97			/			[15]
Triaxial	3.05	5.32	5.91	≤1.81	≤2.94	≤1.43	1000	/	/	[14]
Monoaxial	1.2			≤3			950			[9]
Triaxial	/	/	/	0.05%			192			[21]
Triaxial	0.83	0.38	0.71	4.73, 4.09	2.58, 5.38	7.33, 10.51	1147	1122	2035	This work

### 3.3. Sensor Application in Dynamic Cutting Test

**Figure 11:**
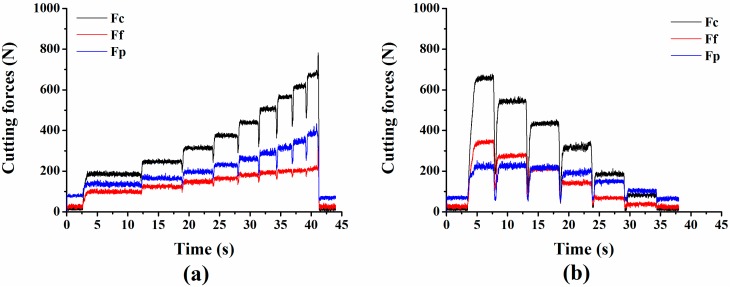
(**a**) Measured cutting force signals in group one; (**b**) Measured cutting force signals in group two.

As described in Figure 11, the measured cutting force signals show a good accordance with the variation of cutting parameters, such as increase in feed rate (group one) and decrease in depth of cut (group two). It is interesting to notice that an abrupt decrease always occurs when cutting parameter changes. This is because adjustment happens when the tool has not completely stepped into the next cutting condition after finishing the last one, so the cutting forces decrease when cutting parameter changes.

**Figure 12:**
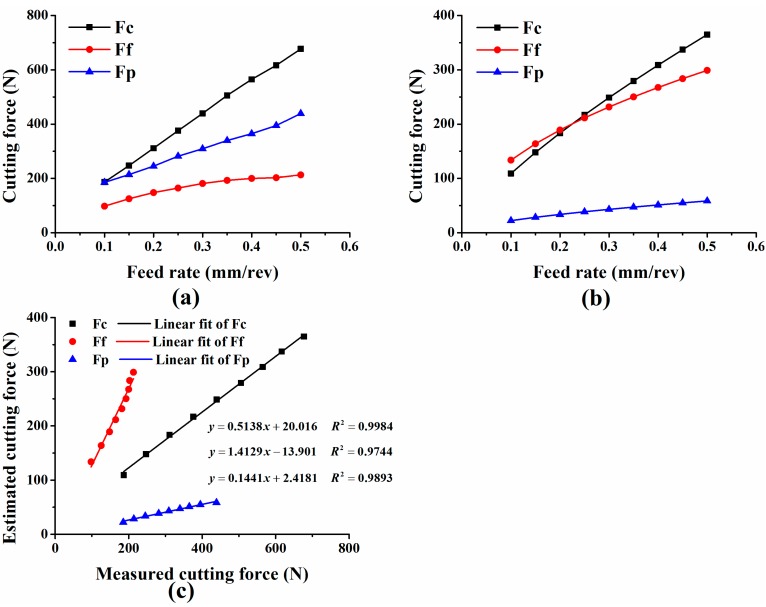
(**a**) Measured cutting forces in group one; (**b**) Estimated cutting forces in group one; (**c**) Correlation analysis between measured and estimated cutting forces.

In order to evaluate the results of the cutting force measurement tests, a comparison between the measured cutting forces and the theoretical trends predicted by empirical model is shown in Figure 12. Figure 12a depicts the measured cutting forces in test group one and Figure 12b shows the estimated cutting forces in group one; where the estimated cutting forces were calculated by Equation (7) and coefficients in Table 3.

Apparently, the estimated cutting forces in Figure 12a differ from the estimated ones in Figure 12b. This does not mean that the developed sensor could not accurately measure cutting forces. Firstly, the measured cutting forces show good agreement with the estimated ones in terms of variation trends; secondly, Equation (7) is an empirical model summarized from practical cutting test, which cannot estimate cutting forces in all conditions accurately. Through correlation analysis between the measured and estimated cutting forces, we find an intensive linear relationship between them as depicted in Figure 12c. This means that a constant multiple-relationship exists between the measured and estimated cutting forces when external noise and measurement error are excluded for consideration. We think the inaccuracy of constant coefficients used in Equation (7) is the main reason for this. As mentioned before, coefficients *K_rFi_*, *K_λFi_*, and *K_γFi_* (*i* *=* *c,f,t*) in Equation (7) are obtained by linear interpolation according to other tool angle coefficients because there are no coefficients for 95° of cemented carbide turning tool in [22]. However, there may be other reasons that affect the difference between the measured and estimated cutting forces, which would be researched in later work. Above all, the measured cutting forces show a good accordance with the variation of cutting parameters in dynamic cutting test, which provides strong evidence for its ability in real-time cutting force measurement.

## 4. Conclusions

This paper presents a new triaxial cutting force sensor for cutting force measurement in high speed metal machining. In order to achieve high accuracy and high natural frequency, a novel structure of two mutual-perpendicular octagonal rings is proposed and three Wheatstone full bridge circuits are organized for measuring triaxial cutting forces. Locations of strain gauges are carefully chosen for limiting cross-interference among cutting force components. Static calibration and impacting modal test result reveal excellent accuracy (≤0.84%) and favorable natural frequency (1147 Hz, 1122 Hz, 2035 Hz in *F_c_*, *F_f_* and *F_p_* directions, respectively) of the developed sensor, which implies that the sensor can meet the demand of cutting force measurement in high precision and high speed metal cutting condition. In dynamic cutting experiment, two groups of cutting tests are implemented and the measured cutting forces display a good accordance with the variation of cutting parameters. Generally, this work achieves the goal of high accuracy and high natural frequency; the devised sensor owns great potential for real-time cutting force measurement. Future work will be focused on cross-interference limitation and dynamic cutting test research.
